# Plant Analysis: A Lecture by Miss Helen C. DeS. Abbott

**Published:** 1887-11

**Authors:** 


### Plant Analysis as an Applied Science. By Helen C. deS. Abbott.

This little pamphlet is the text of a lecture delivered before the Franklin Institute in Philadelphia, January 17th, 1887, and reprinted from the *Journal of the Franklin Institute.*

The lecturer is a young lady well-known in the polite circles of Philadelphia, and celebrated for her scientific acquirements. She has devoted her attention to plant analysis and has made one or two interesting discoveries, especially in the Yucca plant of New Mexico and Arizona. Her object in these investigations is to discover a new generalization that will give a better view—a wider horizon—in the understanding of the relationship of the numerous products obtained from plants, and thus benefit agriculture, materia medica, and many industries that have grown out of the practical uses made of plant products.

The lecture before us is a consideration of the general methods of plant analysis, followed by a description of the great commercial products derived from plants, such as Alcohol, Sugar, Quinine, Alizarine, etc.

On page 4 we find a paragraph of so much value to homœopathists that we quote it:

"Plants which are to be used for medicinal purposes should grow under natural conditions. Cultivation of plants tends to diminish in quantity or to eradicate their noxious or medicinal principles. According to Professor Vogel, hemlock does not yield Coniine in Scotland, cinchona plants are nearly free from Quinine when grown in hothouses and Tannin is also found in the greatest quantity in trees which have a direct supply of sunlight. Wild Belladonna plants contain more alkaloids than the cultivated."

It is our hope that this lady will favor us with another lecture in which will be given an exposition of the new generalization that appears to be growing up out of her interesting and successful work. W. M. J.

